# Regulation of Energy Homeostasis by GPR41

**DOI:** 10.3389/fendo.2014.00081

**Published:** 2014-05-26

**Authors:** Daisuke Inoue, Gozoh Tsujimoto, Ikuo Kimura

**Affiliations:** ^1^Department of Pharmacogenomics, Kyoto University Graduate School of Pharmaceutical Sciences, Kyoto, Japan; ^2^Department of Applied Biological Science, Graduate School of Agriculture, Tokyo University of Agriculture and Technology, Fuchu-shi, Japan

**Keywords:** GPR41, FFAR3, energy regulation, short-chain fatty acid, gut microbiota

## Abstract

Imbalances in energy regulation lead to metabolic disorders such as obesity and diabetes. Diet plays an essential role in the maintenance of body energy homeostasis by acting not only as energy source but also as a signaling modality. Excess energy increases energy expenditure, leading to a consumption of it. In addition to glucose, mammals utilize short-chain fatty acids (SCFAs), which are produced by colonic bacterial fermentation of dietary fiber, as a metabolic fuel. The roles of SCFAs in energy regulation have remained unclear, although the roles of glucose are well-studied. Recently, a G-protein-coupled receptor deorphanizing strategy successfully identified GPR41 (also called free fatty acid receptor 3 or FFAR3) as a receptor for SCFAs. GPR41 is expressed in adipose tissue, gut, and the peripheral nervous system, and it is involved in SCFA-dependent energy regulation. In this mini-review, we focus on the role of GPR41 in host energy regulation.

## Introduction

Dysfunctional energy regulation leads to a variety of metabolic disorders, including obesity (1, 2). Mammals utilize not only glucose as the main energy source, but also short-chain fatty acids (SCFAs), such as acetate, propionate, and butyrate, which are produced by colonic bacterial fermentation of dietary fiber, in a significant proportion of their daily energy requirement (3, 4). The connections between gut microbiota, energy homeostasis, and the pathogenesis of metabolic disorders are now well-established (5, 6). In 2003, several groups reported that two orphan G-protein-coupled receptors (GPCR), namely GPR41 (also called free fatty acid receptor 3 or FFAR3) and GPR43 (also called free fatty acid receptor 2 or FFAR2), are activated by SCFAs (7, 8). GPR41 is reported to couple with Gi/o protein. It is also reported that GPR41 is expressed in adipose tissue, the gut, and the peripheral nervous system. Moreover, GPR41 is reported to be involved in energy regulation in response to SCFAs produced from the gut microbiota. In the following sections, we discuss the role of GPR41 in host energy regulation.

## Adipose Tissue

In adipose tissue, the role of GPR41 in the release of leptin, a polypeptide hormone with pleiotropic effects on appetite and energy metabolism, is the subject of much discussion. *Gpr41* mRNA is known to be expressed in human (7–9) and mouse (10) adipose tissue. Xiong et al. showed that propionate-stimulated activation of GPR41 increases the release of leptin (10). In mice, oral administration of propionate increased plasma leptin levels (10). Furthermore, in experiments using Ob-Luc cells, leptin secretion was increased through overexpression of exogenous *Gpr41* and was decreased by siRNA-mediated knockdown of *Gpr41* (10). Another group showed that propionate-dependent increase in *Leptin* mRNA and protein levels could be inhibited by pretreatment with the Gi/o protein inhibitor, pertussis toxin (9).

However, Hong et al. (11) were unable to detect *Gpr41* mRNA in differentiated 3T3-L1 cells or in mouse white adipose tissue (subcutaneous, perirenal, mesenteric, and epididymal fat pads) (11), even though they used the same PCR primers as Xiong et al. (10). We also previously reported that *Gpr41* expression could not be detected in mouse adipose tissue by quantitative RT-PCR or *in situ* hybridization analysis (12, 13). In contrast, *Gpr43* mRNA, rather than *Gpr41* mRNA, is expressed in mouse adipose tissues (11, 13, 14). Zaibi et al. showed that acetate, rather than butyrate, stimulates leptin secretion by mesenteric adipocytes in wild-type mice (14). GPR41 is activated equally by propionate and butyrate, whereas GPR43 is preferentially activated by propionate rather than butyrate (7). Because of the difference in ligand preference between GPR41 and GPR43, it was suggested that SCFA-stimulated leptin secretion is mediated by GPR43, rather than GPR41 (14). To clarify these discrepancies, the generation of adipose tissue-specific *Gpr41* or *Gpr43* knockout mice will be invaluable.

## Gut

In the gut, GPR41 regulates host energy balance by modulating gut motility. By using *in situ* hybridization analysis, Samuel et al. showed that mouse *Gpr41* mRNA is expressed in cells with the morphologic appearance of enteroendocrine cells (15). The body weight and fat pad weight of *Gpr41* knockout mice are significantly reduced compared to wild-type mice, and this difference is abolished in germ-free conditions (15). These results indicate that the function of GPR41 depends on the SCFA produced by the fermentation of microbiota. Tazoe and colleagues also found the human *Gpr41* is expressed in peptide YY (PYY)-containing enteroendocrine cells (16). Recently, several groups have confirmed *Gpr41* mRNA expression in mouse intestinal L cells, which secrete incretin hormones such as GLP-1 and PYY (17, 18). Samuel and colleagues (15) showed that co-colonization of human gut-derived microbiota in germ-free mice led to significantly increased circulating levels of PYY, which suppresses gut motility. Furthermore, this increase was significantly suppressed in their *Gpr41* knockout littermates, although *Gpr41* deletion did not affect the amount of chow consumption. Intestinal transit rate was significantly faster in *Gpr41* knockout mice compared with wild-type littermates; this phenotype was abolished in germ-free conditions. Moreover, the SCFA content in feces of *Gpr41* knockout mice was significantly higher than in wild-type mice. These results led the authors suggest that the decreased PYY level in *Gpr41* knockout mice increases gut motility, which leads to reduced SCFA absorption and consequently a lean phenotype (15).

In contrast, Bellahcene et al. reported a male-specific increase in body fat mass of *Gpr41* knockout mice when compared to their wild-type littermates; this was observed when mice were fed with either a low- or high-fat diet (19). Deletion of *Gpr41* had no effect on the amount of food intake by either sex, regardless of the type of diet. This included mice of the same age (10 weeks) as those used in the report by Samuel and colleagues (15). The differences in sex hormones could explain why the energy expenditure of female *Gpr41* knockout mice is similar to that of wild-type mice. Nevertheless, it is also possible that reduced SCFA absorption due to increased gut motility is responsible for the alleviation of obesity in *Gpr41* knockout mice (19). Alternatively, reduced energy expenditure in *Gpr41* knockout mice might be caused by reduced sympathetic activity (see Peripheral Nervous System below).

The altered body weight of *Gpr41* knockout mice may be due to differences in genetic backgrounds, or due to the precise constitution of gut microbiota in each animal cohort.

Tolhurst et al. suggested that SCFAs could directly enhance the release of incretin hormones such as GLP-1 and PYY from L cells in gut. In *Gpr41* knockout mice, glucose-stimulated GLP-1 secretion was lower than wild-type mice (17); this was confirmed by Nøhr et al. using the GPR41-selective agonist, AR420626 (18). Consistent with these findings, oral glucose tolerance was impaired in *Gpr41* knockout mice (17).

## Peripheral Nervous System

GPR41 regulates host energy balance by modulating sympathetic activity and intestinal gluconeogenesis. By using *in situ* hybridization and quantitative RT-PCR analysis, we have reported that *Gpr41* mRNA is abundantly expressed in the mouse sympathetic ganglion (12). *Gpr41* knockout mice exhibit a retardation of sympathetic nerve growth. However, further studies will be required to elucidate the precise molecular mechanism by which GPR41 modulates sympathetic nerve differentiation and growth.

In adult wild-type mice, energy expenditure and heart rate are increased by propionate administration; these effects are abolished in *Gpr41* knockout mice (12). The effect of propionate on the heart rate is inhibited by pretreatment with the β-adrenergic receptor blocker propranolol, but not by the nicotinic acetylcholine receptor blocker hexamethonium. These results indicate that propionate activates the sympathetic nervous system (SNS) via GPR41 at the ganglionic level (12). The function of GPR41 in sympathetic ganglia is consistent with the lower energy expenditure and obese phenotype of *Gpr41* knockout mice reported by Bellahcene et al. (19). Furthermore, our laboratory showed that propionate increased the release of norepinephrine from sympathetic neurons through the GPR41–Gβγ–phospholipase C (PLC) β 3-ERK1/2-synapsin 2 (synaptic vesicle-associated phosphoprotein) pathway (12, 20). In addition, we found that β-hydroxybutyrate (β-HB) has a potent antagonistic effect on GPR41 (12). β-HB is a ketone body that can be produced in the liver under ketogenic conditions such as starvation, low-carbohydrate dietary intake, and diabetes. β-HB suppressed propionate-induced sympathetic activation in both primary cultured sympathetic neurons and mice (12, 20). However, acetoacetate, another major ketone body, had no significant effect (12).

Recently, another group demonstrated that SCFA-mediated GPR41 activation improves glucose tolerance by inducing intestinal gluconeogenesis via a gut–brain neural circuit (21). They found *Gpr41* mRNA in the nerve fibers of the portal vein (21). The SCFA-fed rats exhibited improved glucose tolerance compared with standard-diet-fed rats. This effect of SCFA was abolished by portal denervation with capsaicin. Propionate infusion in the portal vein activated jejunum G6Pase, the rate-limiting enzyme for gluconeogenesis. On the other hand, β-HB, an antagonist of GPR41, slightly decreased G6Pase activity when infused alone and reversed propionate-mediated induction of G6Pase (21).

**Figure 1:**
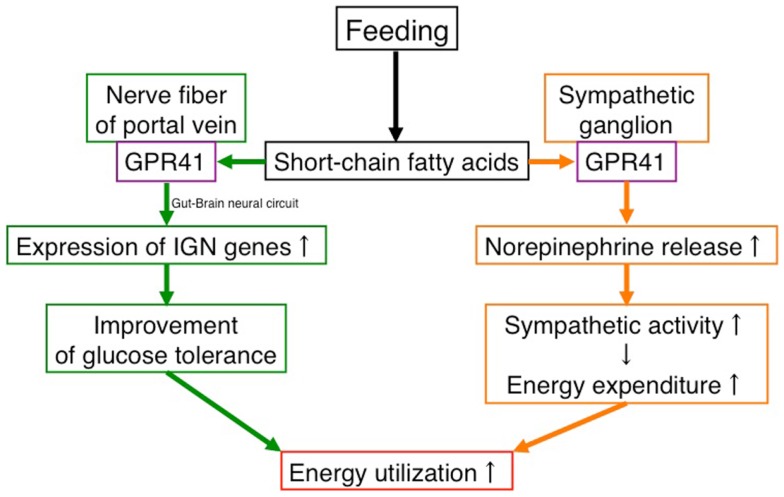
**Effects of SCFAs in energy utilization mediated by GPR41**. Under “fed” conditions, SCFAs are produced in the gut by bacterial fermentation of dietary fiber. SCFAs increase energy utilization by two mechanisms. One is to activate the sympathetic nervous system by stimulating GPR41 in sympathetic ganglia, leading to an increase in energy expenditure. The other is to activate inducing intestinal gluconeogenesis by stimulating GPR41 in the nerve fibers of the portal vein, leading to an improvement of glucose tolerance. In contrast, the β-HB produced in the liver under “fasting” conditions suppresses the activation of GPR41.

These findings suggest that GPR41 functions as an energy sensor in the peripheral nervous system to maintain energy homeostasis (Figure 1).

## Concluding Remarks

It is clear that GPR41 plays a critical role in host energy regulation, although not all of the intracellular signaling cascades that are required for GPR41 function have been elucidated. We envisage that future studies of the interaction between gut microbiota and GPR41, with a particular focus on SCFAs, will provide a more complete picture of GPR41 biological function. Given the beneficial effects that SCFA-dependent GPR41 activation on regulation of metabolism, we suggest that modulating GPR41 by using synthetic ligands will be a promising therapeutic strategy for the treatment of metabolic disorders.

## Conflict of Interest Statement

The authors declare that the research was conducted in the absence of any commercial or financial relationships that could be construed as a potential conflict of interest.
